# High atmospheric CO_2_ concentration causes increased respiration by the oxidative pentose phosphate pathway in chloroplasts

**DOI:** 10.1111/nph.18226

**Published:** 2022-06-15

**Authors:** Thomas Wieloch

**Affiliations:** ^1^ Department of Medical Biochemistry and Biophysics Umeå University Umeå 90187 Sweden

**Keywords:** Calvin–Benson cycle, carbon metabolism, CO_2_ fertilization, glucose‐6‐phosphate shunt, hydrogen stable isotopes, oxidative pentose phosphate pathway, photosynthesis, respiration

## Introduction

Despite significant research efforts, the question of whether rising atmospheric CO_2_ concentration (*C*_a_) affects leaf respiration remains unanswered (Gonzàlez‐Meler *et al*., 2004; Way *et al*., 2015; Dusenge *et al*., 2019). A large body of research conveys an entirely inconsistent picture including reports of both positive and negative responses. This may (*inter alia*) be due to methodological difficulties to disentangle overlapping CO_2_ fluxes at the tissue level and an incomplete understanding of respiration at the metabolic level with a strong research focus on mitochondrial processes. Overall, leaf respiration has remained a major unknown from the metabolic to the Earth system level.

**Fig. 1:**
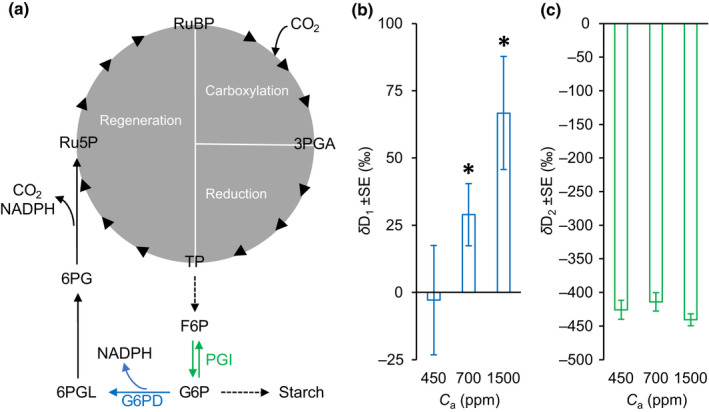
(a) Oxidative pentose phosphate pathway (OPPP) in chloroplasts carrying anaplerotic flux into the Calvin–Benson cycle (grey). Enzyme reactions that introduce deuterium (D) fractionation signals at starch glucose H^1^ and H^2^ are indicated in blue and green, respectively. Dashed arrows, intermediate reactions not shown. The cytosolic oxidative pentose phosphate pathway (not shown) starts from TP that was exported to the cytosol by the triose phosphate translocator (Fliege *et al*., 1978). Synthesized pentose phosphate is reimported into chloroplasts by the pentose phosphate translocator (Eicks *et al*., 2002). Enzymes: G6PD, glucose‐6‐phosphate dehydrogenase; PGI, phosphoglucose isomerase. Metabolites: 3PGA, 3‐phosphoglycerate; 6PG, 6‐phosphogluconate; 6PGL, 6‐phosphogluconolactone; F6P, fructose 6‐phosphate; G6P, glucose 6‐phosphate; NADPH, nicotinamide adenine dinucleotide phosphate; Ru5P, ribulose 5‐phosphate; RuBP, ribulose 1,5‐bisphosphate; TP, triose phosphates (glyceraldehyde 3‐phosphate, dihydroxyacetone phosphate). Modified figure from Wieloch *et al*. (2022b). (b, c) D abundance at glucose H^1^ (blue bars) and H^2^ (green bars) of sunflower leaf starch. Asterisks denote D abundances that are significantly greater than zero (one‐tailed one‐sample *t*‐test: *P* < 0.05, *n* = 5). The plants were raised in chambers over 7–8 wk at an atmospheric CO_2_ concentration (*C*_a_) of 450 ppm. After a day in darkness to drain the starch reserves, the plants were grown for 2 d at different levels of *C*_a_ (450, 700, 1500 ppm) corresponding to different levels of intercellular CO_2_ concentration (328, 531, 1365 ppm). Data expressed as *δ*D_1_ = D_1_/D_6S_ − 1 and *δ*D_2_ = D_2_/D_6R_ – 1, where D_*i*_ denotes relative D abundances at specific carbon‐bound hydrogen atoms of glucose. D abundances at glucose H^6S^ and H^6R^ are used as references because glucose H^1^ and H^6S^ and H^2^ and H^6R^ have the same precursors at the chloroplast triose‐phosphate level, and H^6S^ and H^6R^ are not modified in the starch biosynthesis pathway (Wieloch *et al*., 2022a).

State‐of‐the‐art isotope techniques enable analyses of specific metabolic fluxes (Ehlers *et al*., 2015; Wieloch *et al*., 2021b, 2022c; Xu *et al*., 2022). Recently, we reported two deuterium (D) fractionation signals (i.e. systematic variability in D abundance) in starch glucose of sunflower leaves (Wieloch *et al*., 2022a). A signal at glucose H^1^ reflects hydrogen (H) isotope fractionation by chloroplast glucose‐6‐phosphate dehydrogenase (G6PD) and associated flux through the oxidative pentose phosphate pathway (OPPP; Fig. 1a). This anaplerotic pathway feeds pentose phosphates into the Calvin–Benson cycle (CBC), supplies NADPH, and releases CO_2_. Another signal at glucose H^2^ reflects H isotope fractionation by chloroplast phosphoglucose isomerase (PGI) and associated shifts of the reaction catalysed by PGI from kinetic to equilibrium conditions.

Here, these fractionations at glucose H^1^ and H^2^ are respectively expressed as:

(Eqn 1) $$\delta D_{1}=\frac{D_{1}}{D_{6S}}-1$$

and

(Eqn 2) $$\delta D_{2}=\frac{D_{2}}{D_{6R}}-1$$

where D_*i*_ denotes relative D abundances at specific carbon (C)‐bound H atoms of glucose. In these equations, D abundances at glucose H^6S^ and H^6R^ are used as references because glucose H^1^ and H^6S^ and H^2^ and H^6R^ have the same precursors at the chloroplast triose‐phosphate level, and H^6S^ and H^6R^ are not modified in the starch biosynthesis pathway (Wieloch *et al*., 2022a). In these notations, increases of *δ*D_1_ above zero reflect increases in anaplerotic flux into the CBC, whereas increases of *δ*D_2_ from negative to positive values reflect shifts of the PGI reaction from being on the side of fructose 6‐phosphate (F6P), to being at equilibrium, to being on the side of glucose 6‐phosphate (G6P) (Wieloch *et al*., 2022a).

Previously, we investigated these processes in leaves of sunflowers raised over 7–8 wk at *C*_a_ = 450 ppm (Wieloch *et al*., 2022a). We reported evidence against anaplerotic flux under these conditions. However, moving the plants into a low‐*C*_a_ atmosphere for 2 d led to significant increases in *δ*D_1_ and *δ*D_2_, consistent with an increase in anaplerotic flux and a shift of the PGI reaction from kinetic to equilibrium conditions, respectively (see reanalysis of previous low‐*C*_a_ results based on Eqns S1 and S2 in Supporting Information Notes S1). Related fractionation signals were also found in the starch derivative tree‐ring glucose under drought (Wieloch *et al*., 2018, 2022b).

Here, I reanalyse our previously published data (Wieloch *et al*., 2022a) to assess how metabolism behaves after moving the plants into a high‐*C*_a_ atmosphere for 2 d. I report that, as *C*_a_ increases from 450 to 1500 ppm, respiration by the OPPP in chloroplasts increases from 0 to ≈ 5% relative to the rate of net C assimilation. This is consistent with known regulatory properties of the pathway. Summarizing recent reports of metabolic fluxes in plant leaves, a picture emerges in which mitochondrial processes seem distinctly less important for overall respiration than the OPPPs in chloroplasts and the cytosol. My findings and regulatory properties of these pathways are consistent with observations of lower than expected increases of photosynthesis in response to increasing *C*_a_. Reported advances in understanding leaf respiratory mechanisms may enable modelling and prediction of respiration effects (*inter alia*) on biosphere–atmosphere CO_2_ exchange and plant performance under climate change.

## Anaplerotic flux and associated respiration increase at high *C*_a_

In contrast to *C*_a_ = 450 ppm, *δ*D_1_ is significantly greater than zero at *C*_a_ = 700 ppm (29‰) and *C*_a_ = 1500 ppm (67‰) (Fig. 1b; one‐tailed one‐sample *t*‐test: *P* < 0.05, *n* = 5). This is consistent with significant anaplerotic flux into the CBC. By contrast, *δ*D_2_ exhibits low values of *c*. −427‰ at *C*_a_ ≥ 450 ppm (Fig. 1c), indicating that the PGI reaction remains stably removed from equilibrium on the side of F6P (cf. Wieloch *et al*., 2022a). The absence of a *δ*D_2_ response is remarkable, because anaplerotic flux was proposed to be controlled at the level of PGI (Sharkey & Weise, 2016). Accordingly, we previously observed simultaneous shifts of *δ*D_1_ and *δ*D_2_ for *C*_a_ shifts below 450 ppm (Fig. S1) (Wieloch *et al*., 2022a). Thus, the results suggest regulatory differences of the anaplerotic pathway for low and high *C*_a_ conditions.

In the light, chloroplast G6PD is inhibited by redox regulation via thioredoxin (Née *et al*., 2009), yet inhibition may be reversed allosterically by increasing concentrations of G6P (Cossar *et al*., 1984; Preiser *et al*., 2019). At medium *C*_a_, the PGI reaction in chloroplasts is strongly removed from equilibrium on the side of F6P, resulting in low [G6P]/[F6P] ratios and G6P concentrations (Dietz, 1985; Gerhardt *et al*., 1987; Kruckeberg *et al*., 1989; Schleucher *et al*., 1999). Low G6P concentrations are believed to restrict the anaplerotic flux (Sharkey & Weise, 2016). Towards low *C*_a_, G6P concentrations increase more than F6P concentrations, that is, the PGI reaction shifts towards equilibrium (Dietz, 1985). Towards high *C*_a_, [G6P]/[F6P] ratios remain low, yet F6P and G6P concentrations both increase along with net C assimilation (Dietz, 1985). Thus, towards low *C*_a_, G6P concentrations and anaplerotic flux increase due to regulation at PGI. By contrast, increases towards high *C*_a_ are not caused by regulation at PGI but probably by increases in net C assimilation and concomitantly increasing G6P concentrations.

## Estimation of anaplerotic flux and associated respiration at high *C*_a_

A previously published model describing H isotope fractionation by G6PD can be used to estimate anaplerotic flux into the CBC, associated respiration, and NADPH supply (Wieloch *et al*., 2022a). At *C*_a_ = 700 ppm, ≈ 4.2% of the G6P entering the starch biosynthesis pathway is diverted into the anaplerotic pathway, whereas it is ≈ 9.4% at 1500 ppm. Assuming 50% of all net assimilated C becomes starch (Sharkey *et al*., 1985), anaplerotic respiration proceeds at ≈ 2% and ≈ 5% relative to net C assimilation at *C*_a_ = 700 ppm and 1500 ppm, respectively. These estimates are based on *δ*D_1_ signal strengths in starch glucose. At medium to high *C*_a_, the PGI reaction is on the side of F6P (Fig. 1c) (Dietz, 1985). To a degree, this prevents conversion of G6P (the site of signal introduction) back to F6P. F6P may leave the starch biosynthesis pathway via transketolase, causing signal washout (Wieloch *et al*., 2022a). At low *C*_a_, the PGI reaction is closer to or at equilibrium and signal washout can be expected to be significant (Notes S1) (Wieloch *et al*., 2022a). Thus, the G6PD fractionation model may significantly underestimate anaplerotic flux at low *C*_a_, whereas high‐*C*_a_ estimates can be expected to be closer to actual values.

## Potential causes of lower than expected increases of photosynthesis in response to increasing *C*_a_

In C_3_ plants, net C assimilation increases with increasing *C*_a_ (Drake *et al*., 1997; Ainsworth & Long, 2005). However, responses seen in free‐air CO_2_ enrichment (FACE) experiments differ significantly among plant functional groups, with trees showing the strongest increase (Nowak *et al*., 2004; Ainsworth & Long, 2005). Hence, some plant functional groups apparently come closer to theoretically possible increases calculated from Rubisco kinetics than others do (Long, 1991). As already shown herein, anaplerotic flux increases with increasing *C*_a_. Thus, respiration by the anaplerotic pathway can explain part of the lower than expected increase of net C assimilation by *C*_a_.

**Fig. 2:**
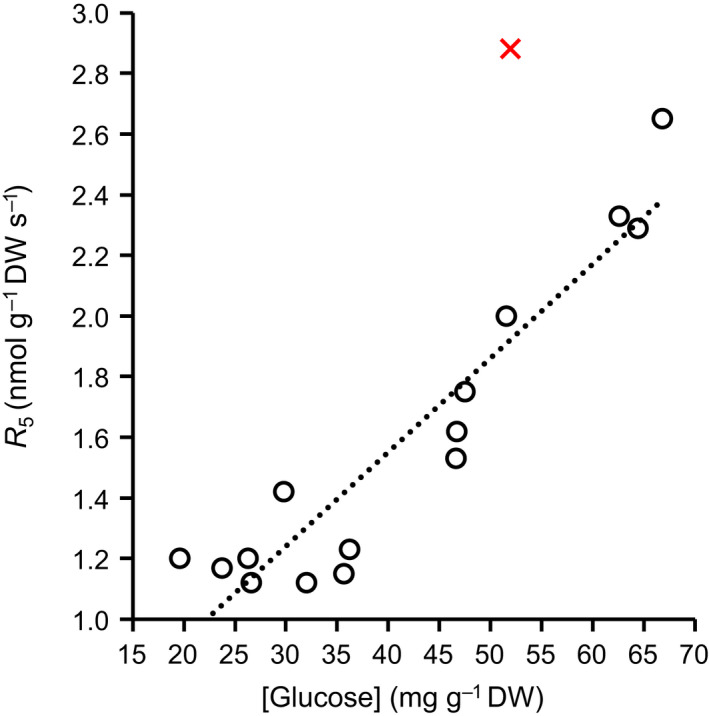
Respiration at 5°C (*R*_5_) as a function of glucose concentration in illuminated needles of 33‐yr‐old *Pinus banksiana*. Dotted line, positive linear relationship between both variables (*R* ^2^ = 0.88, *P* < 10^−6^, *n* = 15). Red cross, outlier removed before regression analysis. Data collected from eight provenances (boreal to temperate origin, 44–55°N) grown in a common garden in Cloquet, MN, USA. Sun‐exposed canopy branch sampled from four randomly selected trees per provenance at two dates, one in mid‐November 1997 and one in mid‐May 1998. *R*_5_ measured in a laboratory at an atmospheric CO_2_ concentration of *c*. 380 ppm within 6 h after sampling by infrared gas analysers and cuvettes (LCA‐3 and PLC‐C; Analytical Development Co., Hoddesdon, UK). Note, respiration was shown to remain stable over several hours after sampling (Mitchell *et al*., 1999; Ow *et al*., 2008; Tjoelker *et al*., 2009). Glucose concentration measured using a high‐performance liquid chromatograph (Waters Associates, Milford, MA, USA) equipped with a Sugar Pack I column and a refractive index detector (Waters 410) following published procedures (Pukacka & Pukacki, 1997). Soluble sugar extracted from dried needles used as starting material. Figure shows data published by Tjoelker *et al*. (2009). For further information on materials and methods, see Tjoelker *et al*. (2009).

Another part may be explained by respiration by the cytosolic OPPP. Primary control of flux through this pathway is exerted at the level of its first enzyme, G6PD. In the light, cytosolic G6PD activity in potato leaf discs was shown to increase with increasing glucose concentration through *de novo* enzyme synthesis (Hauschild & von Schaewen, 2003). In Arabidopsis rosettes, 88% of the glucose was shown to be in the vacuole and cytosol (Szecowka *et al*., 2013). As part of sucrose cycling, cytosolic hexokinase converts this glucose into G6P (Dancer *et al*., 1990; Xu *et al*., 2022). G6P‐derived C can re‐enter the CBC via the cytosolic OPPP (Eicks *et al*., 2002; Xu *et al*., 2022). Combinedly, sucrose cycling and flux through the cytosolic OPPP can explain ^13^C labelling lags of CBC metabolites (Sharkey *et al*., 2020; Xu *et al*., 2022). These workers estimate that respiration by the cytosolic OPPP proceeds at ≈ 5% relative to the rate of net C assimilation in poplar at 30°C and in camelina at 22°C. Since ^13^C labelling lags of CBC metabolites appear to occur generally (Mahon *et al*., 1974; Canvin, 1979; Hasunuma *et al*., 2010; Szecowka *et al*., 2013; Ma *et al*., 2014; Sharkey *et al*., 2020; Xu *et al*., 2022), and since, by supplying cytosolic NADPH, the OPPP fulfils an important physiological function (Wieloch & Sharkey, 2022), substantial OPPP respiration is likely a general feature of C_3_ plants. Previously, Tjoelker *et al*. (2009) reported a positive linear relationship between respiration at 5°C and leaf soluble‐sugar concentration in illuminated pine needles (*R* ^2^ = 0.49, *P* < 10^−4^, *n* = 40). In their study, soluble sugar concentration denotes the average concentration of raffinose, sucrose, glucose, and fructose. Reanalysing the data of Tjoelker *et al*. (2009), I find a positive linear relationship between respiration at 5°C and glucose concentration (Fig. 2; *R* ^2^ = 0.88, *P* < 10^−6^, *n* = 15). Leaf soluble‐sugar concentration generally increases with increasing *C*_a_ (Ainsworth & Long, 2005). Taken together, these data may suggest that, as *C*_a_ increases, leaf glucose concentration increases, causing increased expression of cytosolic G6PD and flux through the cytosolic OPPP. Associated respiration may explain part of the lower than expected increase of net C assimilation in response to increasing *C*_a_.

Compared with other plant functional groups, trees exhibit stronger increases in net C assimilation in response to increasing *C*_a_ (Ainsworth & Long, 2005) and are thus getting closest to theoretically possible increases (Long, 1991). However, the additional C does not result in stronger increases in leaf soluble‐sugar concentration. On the contrary, trees exhibit lower increases in leaf soluble‐sugar concentration than other plant functional groups do (Ainsworth & Long, 2005). By contrast, trees exhibit the highest increase in dry matter accumulation (Ainsworth & Long, 2005). Thus, low increases in leaf soluble‐sugar concentration are probably explained by high sink strengths of the relatively young and fast‐growing trees studied in FACE experiments. In turn, low increases in leaf soluble‐sugar concentration may cause low increases in respiration by both the plastidial and cytosolic OPPP (see earlier). This may explain why trees come closer to theoretically possible increases in net C assimilation in response to increasing *C*_a_ than other plant functional groups do. Overall, based on analyses and argumentation presented here, increases of net C assimilation by increasing *C*_a_ (including acclimation effects) may depend on plant sink strength and associated OPPP respiration.

OPPP flux in chloroplasts introduces a *δ*D_1_ signal in starch (see earlier). Similarly, OPPP flux in the cytosol of leaves can be expected to introduce a *δ*D_1_ signal in the glucosyl and fructosyl moieties of sucrose. These signals will be recorded in tree‐ring cellulose because tree‐ring cellulose is synthesized from starch and sucrose. However, signal interpretation will be complicated by several processes, including signal washout in chloroplasts (see earlier) and OPPP flux and triose phosphate cycling in the cytosol of tree‐ring cells. Nevertheless, I believe these complications can be addressed, and I encourage the development of *δ*D_1_ signal analysis to retrieve information about leaf respiration and respiratory acclimation to increasing *C*_a_ from leaf, phloem, and tree‐ring metabolites.

## A paradigm shift in the field of leaf day respiration?

In the past, research of leaf day respiration had a strong focus on mitochondrial processes. However, according to recent analyses of metabolic fluxes in leaves of *Arabidopsis thaliana* and *Camelina sativa*, mitochondrial respiration is relatively low (1–1.6% relative to the rate of net C assimilation) (Ma *et al*., 2014; Xu *et al*., 2022). By contrast, respiration by the cytosolic OPPP is ≈ 5% relative to the rate of net C assimilation in *C. sativa* leaves (Xu *et al*., 2022). Similarly, in sunflower leaves, respiration by the OPPP in chloroplasts is relatively high under both high (see earlier herein) and low *C*_a_ (Notes S1; Wieloch *et al*., 2021a, 2022a). These findings indicate that the OPPP may be more important for overall leaf day respiration than mitochondrial processes.

## Competing interests

None declared.

## Supporting information


**Fig. S1** Deuterium abundance at glucose H^1^ and H^2^ of sunflower leaf starch.
**Notes S1** Recalculation of previously reported estimates of flux through the plastidial anaplerotic pathway at low *C*_a_.
Please note: Wiley Blackwell are not responsible for the content or functionality of any Supporting Information supplied by the authors. Any queries (other than missing material) should be directed to the *New Phytologist* Central Office.

## Data Availability

The data supporting the findings of this study have been published previously (Tjoelker *et al*., 2009; Wieloch *et al*., 2022a).
