# Supplementary material for: High atmospheric CO2 concentration causes increased respiration by the oxidative pentose phosphate pathway in chloroplasts
Source: New Phytol. 2022 Jun 15;235(4):1310–4. doi: 10.1111/nph.18226 (PMC9546095; doi:10.1111/nph.18226)
Supplement: Supplementary file 1 — Fig. S1 Deuterium abundance at glucose H1 and H2 of sunflower leaf starch. Notes S1 Recalculation of previously reported estimates of flux through the plastidial anaplerotic pathway at low C a. Please note: Wiley Blackwell are not responsible for the content or functionality of any Supporting Information supplied by the authors. Any queries (other than missing material) should be directed to the New Phytologist Central Office. [file NPH-235-1310-s001.pdf]

**High atmospheric $CO_2$ concentration causes increased respiration by the oxidative pentose phosphate pathway in chloroplasts – Supporting information (Accepted: 8 May 2022)**

Thomas Wieloch

## Notes S1. Recalculation of previously reported estimates of flux through the plastidial anaplerotic pathway at low $C_a$

In Wieloch *et al.* (2022), we expressed fractionation signals discussed here as

$$\delta \mathrm{D}_i = \frac{\mathrm{D}_i}{\Sigma \mathrm{D}_{\mathrm{ME}}/6} - 1 \qquad \text{Eqn S1}$$

where $D_i$ and $D_{ME}$ denote relative deuterium abundances at specific carbon-bound hydrogens of glucose and the six methyl-group hydrogens of the glucose derivative used for NMR measurements (3,6-anhydro-1,2-*O*-isopropylidene-α-D-glucofuranose), respectively. Here, I express these signals as

$$\delta \mathrm{D}_1 = \frac{\mathrm{D}_1}{\mathrm{D}_{6S}} - 1 \qquad \text{Eqn S2}$$

and

$$\delta \mathrm{D}_2 = \frac{\mathrm{D}_2}{\mathrm{D}_{6R}} - 1 \qquad \text{Eqn S3}$$

Based on equation S2, $\delta D_1$ is 66‰ at $C_a$ = 280 ppm and 92‰ at $C_a$ = 180 ppm (Figure S1A). Furthermore, $\delta D_1$ and thus flux through the plastidial anaplerotic pathway is significantly greater than zero at $C_a$ = 180 ppm ($p < 0.05$, $n = 2$) while it comes close to being significantly greater than zero at $C_a$ = 280 ppm ($p < 0.13$, $n = 2$). A previously published model describing deuterium fractionation by G6PD can be used to estimate the plastidial anaplerotic flux, and associated respiration (Wieloch *et al.*, 2022). Based on this model, ≈ 9.3% and 12.7% of the G6P entering the starch biosynthesis pathway is diverted into the anaplerotic pathway at $C_a$ = 280 and 180 ppm, respectively. Assuming 50% of all net assimilated carbon becomes starch (Sharkey *et al.*, 1985), anaplerotic flux and associated respiration proceeds at ≈ 5% and ≈ 7%

relative to the rate of net carbon assimilation at $C_a$ = 280 and 180 ppm. These rates are probably strongly underestimated since, at low $C_a$, much of the fractionation signal introduced by G6PD can be expected to not arrive in starch (see biochemical explanation in Wieloch *et al.*, 2022).

Based on equation S3, $\delta D_2$ is ≈ -427‰ at $C_a \geq$ 450 ppm, -273‰ at $C_a$ = 280 ppm, and -15‰ at $C_a$ = 180 ppm (Figure S1B). This indicates that the PGI reaction is on the side of F6P at $C_a \geq$ 450 ppm and shifts towards equilibrium with decreasing $C_a$ below 450 ppm (Wieloch *et al.*, 2022).

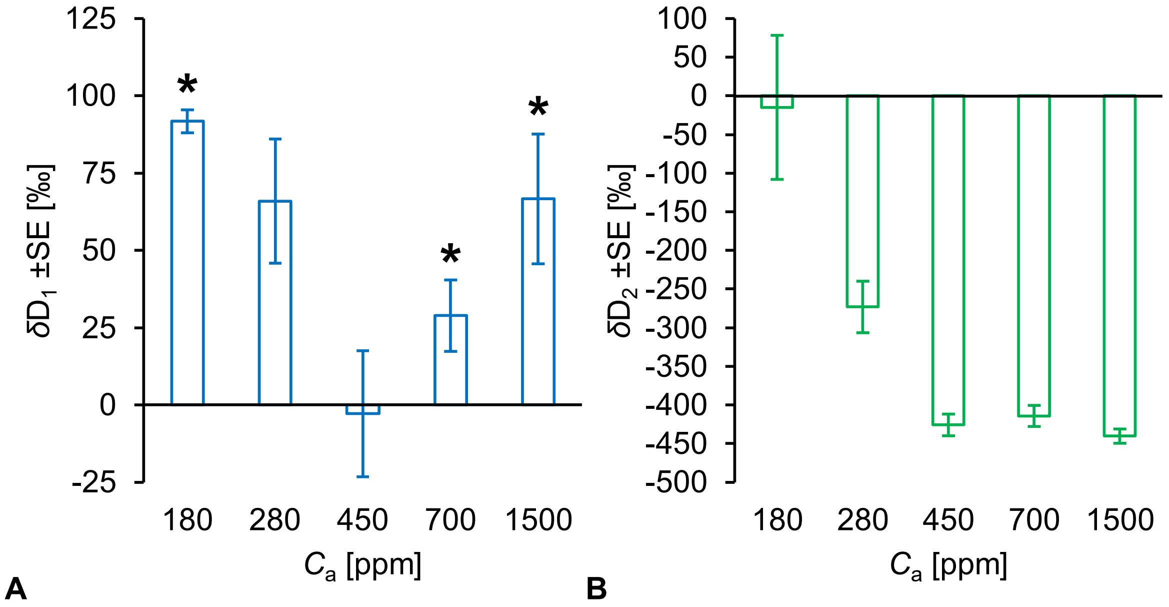


**Figure S1** Deuterium abundance at glucose H[1] (**A**, blue bars), and H[2] (**B**, green bars) of sunflower leaf starch. Asterisks denote deuterium abundances that are significantly greater than zero (one-tailed one-sample t-test: $p < 0.05$, $n \geq 2$). At 280 ppm, $\delta D_1$ is close to being significantly greater than zero ($p < 0.13$, $n = 2$). The plants were raised in chambers over 7 to 8 weeks at $C_a$ = 450 ppm. After a day in darkness to drain the starch reserves, the plants were grown for two days at different levels of $C_a$ (180, 280, 450, 700, 1500 ppm) corresponding to different levels of $C_i$ (140, 206, 328, 531, 1365 ppm). Data expressed as $\delta D_1 = D_1/D_{6S} - 1$ and $\delta D_2 = D_2/D_{6R} - 1$ where $D_i$ denotes relative deuterium abundances at specific carbon-bound hydrogens of glucose. Deuterium abundances at glucose $H^{6S}$ and $H^{6R}$ are used as references because glucose $H^1$ and $H^{6S}$ and $H^2$ and $H^{6R}$ have the same precursors at the chloroplast triose-phosphate level, and $H^{6S}$ and $H^{6R}$ are not modified in the starch biosynthesis pathway (Wieloch *et al.*, 2022).

## References

**Sharkey TD, Berry JA, Raschke K**. **1985**. Starch and sucrose synthesis in *Phaseolus vulgaris* as affected by light, $CO_2$, and abscisic acid. *Plant Physiology* **77**: 617–620.

**Wieloch T, Augusti A, Schleucher J**. **2022**. Anaplerotic flux into the Calvin-Benson cycle. Hydrogen isotope evidence for *in vivo* occurrence in $C_3$ metabolism. *New Phytologist* **234**: 405–411.
